# The effectiveness of PD-1 inhibitors in non-small cell lung cancer (NSCLC) patients of different ages

**DOI:** 10.18632/oncotarget.23678

**Published:** 2017-12-26

**Authors:** Yingcheng Wu, Qianqian Ju, Bei Qian, Feng Zhang, Hui Shi

**Affiliations:** ^1^ Medical School of Nantong University, Jiangsu 226001, China; ^2^ Laboratory Animal Center of Nantong University, Jiangsu 226001, China; ^3^ School of Nursing, Nantong University, Jiangsu 226001, China; ^4^ Department of Thoracic Surgery, Affiliated Hospital of Nantong University, Jiangsu 226001, China

**Keywords:** PD-1 inhibitor, age, meta-analysis, overall survival, progression-free survival

## Abstract

**Background:**

Immunosenescence, the age-related decline of immunity, affects the immune responses of non-small cell lung cancer (NSCLC) patients. Through immune responses, programmed death-1 (PD-1) inhibitors exert their antitumor robustness. In different ages of NSCLC patients, especially the older patients, the effectiveness of PD-1 inhibitors remains unclear. It is still controversial whether pembrolizumab or nivolumab should be used in treating NSCLC patients.

**Results:**

2,192 NSCLC patients from four phase III RCTs were included. PD-1 inhibitors significantly prolonged the OS in both younger group (<65-year-age) (HR: 0.64, 95% CI: 0.54–0.75, *P* = 0.000) and older group (≥65-year-age) (HR: 0.68, 95% CI: 0.54–0.81, *P* = 0.001) than chemotherapy. Among patients aged over 75, no significantly longer OS was observed (HR: 1.02, 95% CI: 0.35–1.69, *P* = 0.971) than controls. In the older group (≥65-year-age), HR of OS favors nivolumab rather than pembrolizumab.

**Conclusions:**

Among patients aged over 75, no significantly prolonged overall survival was observed compared with chemotherapy. In comparison with pembrolizumab, nivolumab was associated with better OS in older NSCLC patients (≥65-year-age), and better PFS in all NSCLC patients. Older patients, especially those aged over 75, should be paid more attention to in the future clinical trials, guidelines, and clinical practice.

**Methods:**

The authors included clinical trials testing PD-1 inhibitors (nivolumab and pembrolizumab) compared with chemotherapies in older and younger patients. The authors used the hazard ratio (HR) and 95% confidence interval (CI) of overall survival (OS) and progression-free survival (PFS).

## INTRODUCTION

The progress of immunotherapy, especially the programmed cell death protein-1 (PD-1) inhibitors, has created a major paradigm shift within the therapeutic landscape of several solid tumors [1–3]. The United States Food and Drug Administration's approval of nivolumab and pembrolizumab had brought hope to the patients and the scientific community [4, 5].

In the United States, the incidence of lung cancer was higher in older people based on a 2016 report, and mortality correlated with age of patients [6]. In particular, for patients aged 80–84, the incidence and mortality reached the peak. The median age at diagnosis of lung was about 70 years. Furthermore, in the clinic, the efficacy and toxicity of chemotherapy was associated with age of non-small cell lung cancer (NSCLC) patients [7]. In the field of immune checkpoint inhibitors (ICIs), it was observed that effectiveness varies between younger and older patients [8]. Similar phenomena were depicted among melanoma patients [9], urothelial carcinoma patients [10], and renal-cell carcinoma patients [11]. Notably, previous research manifested that patients aged more than 75 years might not benefit from immune checkpoint inhibitors [8].

Immunosenescence, which refers to the age-related decline of immunity, affected the survival of NSCLC patients [12]. From a preclinical perspective, functions of antigen-presenting cells (APCs) altered during immunosenescence [13]. Also, senescent T cells were demonstrated to have a reduced ability to eliminate tumor cells [12]. Since PD-1 inhibitors exert their antitumor robustness through T cells, chances are that immunosenescence can have a negative influence on the effectiveness in aged patients.

According to previous clinical trials, whether the aged NSCLC patients could benefit from PD-1 inhibitors remained controversial. In older people, little is known whether pembrolizumab or nivolumab should be used. A previous study compared the effectiveness of different ICIs in younger and older cancer patients in general, however, the researchers did not give recommendations for NSCLC patients specifically because of the lack of published data [8]. Hence, we conducted this meta-analysis in order to compare the PD-1 inhibitors in younger and older patients, taking into accounts the best choice of PD-1 inhibitor for the aged people.

## RESULTS

### Search results and population characteristics

In all, 2,192 NSCLC patients from four phase III randomized controlled trials (RCTs) were included in the analysis [15–18]. The trial selection process was illustrated in Supplementary Figure 1. All the trials are phase III RCTs. All the studies assessed the efficacy of PD-1 inhibitors (*n* = 1,271) versus chemotherapies (*n* = 921). The main characteristics of all the trials were available in Table 1.

**Table 1:** Characteristics of the studies included in the meta-analysis

First author, year	Phase	Type of NSCLC	Arm	No. of patients for analysis	Age (years) median (range)
Reck 2016	III	Advanced NSCLC	Pembrolizumab 200 mg	154	64.5 (33–90)
			ICC	151	66.0 (38–85)
Herbst 2016	II/III	Advanced NSCLC	Pembrolizumab 2 mg/kg	344	63·0 (56·0–69·0)
			Pembrolizumab 10 mg/kg	346	63·0 (56·0–69·0)
			Docetaxel	343	62·0 (56·0–69·0)
Borghaei 2015	III	Advanced nonsquamous NSCLC	Nivolumab 3 mg/kg	292	61 (37–84)
			Docetaxel	290	64 (21–85)
Brahmer 2015	III	Advanced squamous NSCLC	Nivolumab 3 mg/kg	135	62 (39–85)
			Docetaxel	137	64 (42–84)

ICC, investigator's choice chemotherapy; NSCLC, non–small-cell lung cancer.

### Primary outcome: overall survival

1,887 patients from three RCTs were included in the OS analysis [16–18]. Two RCTs divided patients into three subgroups by age, including <65-, ≥65 to <75, and ≥75-year-old groups [17, 18]. Nevertheless, one RCT reported two subgroups by using 65-year-old as a cut-off age [16]. Hence, we combine the two groups (≥65 to <75-year-old group and ≥75-year-old group) into one group. In addition, we analyze the overall survival in patients older than 75 particularly (*n* = 72). The fixed-effect model was applied because there was a low heterogeneity (*I*^2^ = 0.1%) in the analysis.

**Figure 1:**
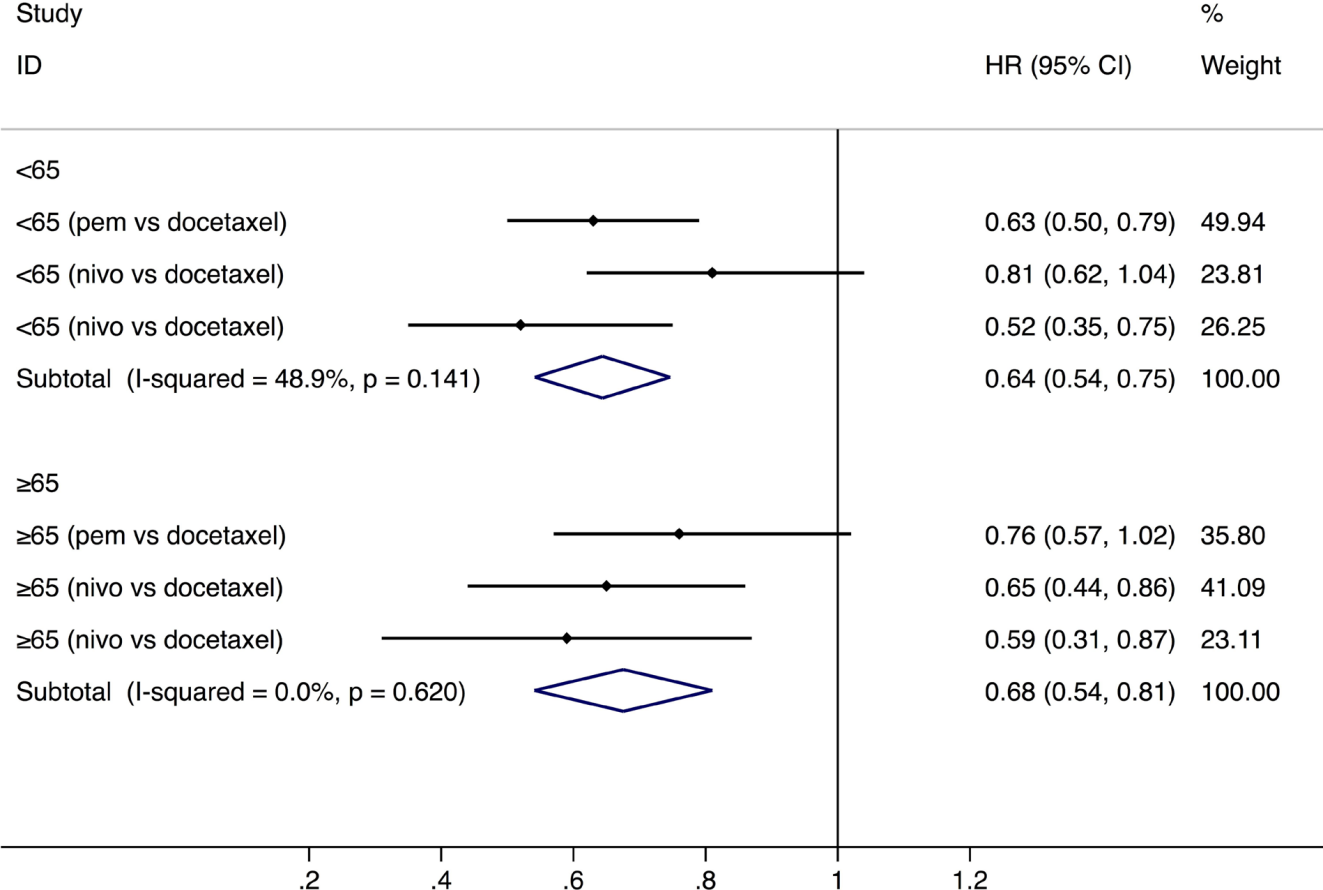
Comparison of overall survival between younger and older groups with a cut-off age of 65 Abbreviations: nivo, nivolumab; pem, pembrolizumab; HR, hazard ratio; CI, confidence interval.

**Figure 2:**
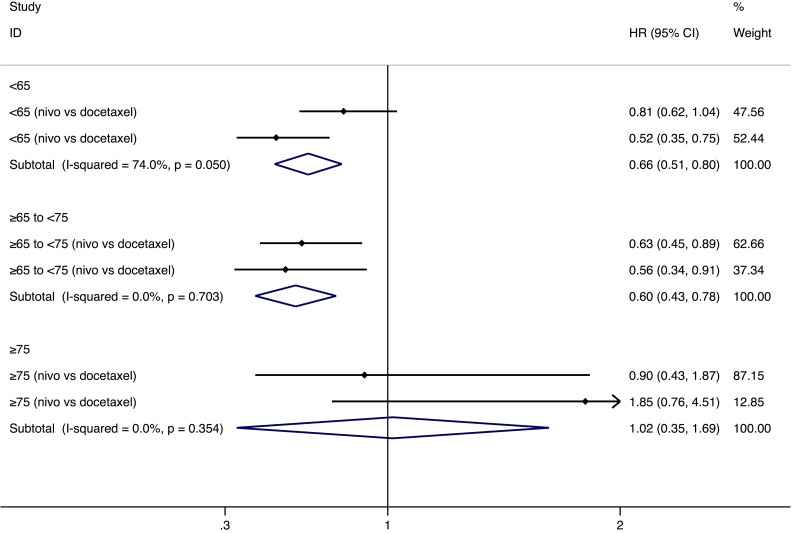
Comparison of overall survival between different ages Abbreviations: nivo, nivolumab; pem, pembrolizumab; HR, hazard ratio; CI, confidence interval.

PD-1 inhibitors significantly prolonged the OS in both younger group (<65-year-age) (HR: 0.64, 95% CI: 0.54–0.75, *P* = 0.000, *n* = 1,095) and older group (≥65-year-age) (HR: 0.68, 95% CI: 0.54–0.81, *P* = 0.001, *n* = 792) than chemotherapy (Figure 1). The analysis based on three age-subgroups (<65-, ≥65 to <75-year-old group, and ≥75-year-old group) manifested that, among patients older than 75 years old, no significantly longer OS was observed (HR: 1.02, 95% CI: 0.35–1.69, *P* = 0.971) (Figure 2). Subgroup analysis by the type of PD-1 inhibitors (nivolumab and pembrolizumab) was also conducted (Table 2). In the older group (≥65-year-age), HR of OS favors nivolumab (Table 2).

**Table 2:** Subgroup analysis by the type of PD-1 inhibitors

Age	Type of PD-1 inhibitors	Outcome	HR (95% CI)	*P* value
<65	Pembrolizumab	OS	0.63 (0.50, 0.79)	<0.001
<65	Nivolumab	OS	0.66 (0.51, 0.80)	<0.001
≥65	Pembrolizumab	OS	0.76 (0.53, 0.98)	<0.001
≥65	Nivolumab	OS	0.63 (0.46, 0.80)	<0.001
<65	Pembrolizumab	PFS	0.75 (0.53, 0.97)	<0.001
<65	Nivolumab	PFS	0.62 (0.40, 0.85)	<0.001
≥65	Pembrolizumab	PFS	0.69 (0.22, 1.16)	0.004
≥65	Nivolumab	PFS	0.54 (0.29, 0.79)	<0.001

### Secondary outcome: progression-free survival

**Figure 3:**
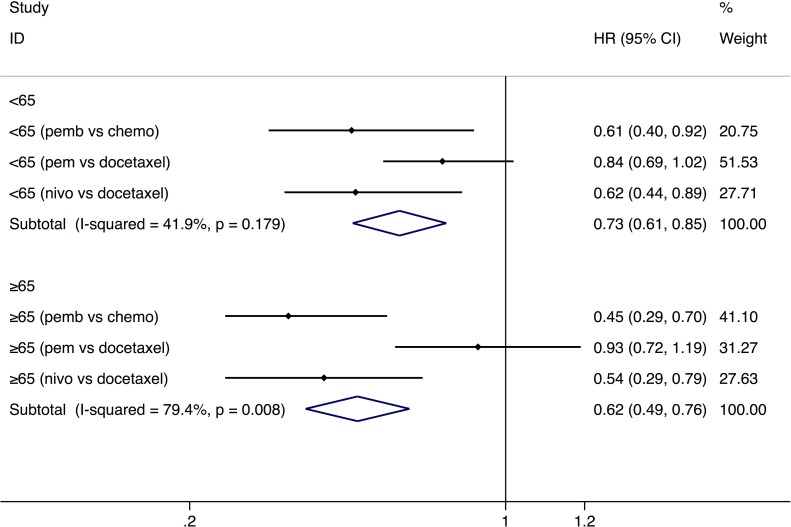
Comparison of progression-free survival between younger and older groups with a cut-off age of 65 Abbreviations: nivo, nivolumab; pem, pembrolizumab; HR, hazard ratio; CI, confidence interval.

The PFS analysis was based on four trials, which enrolled a total of 1,610 patients [15, 16, 18]. A study reported PFS result separately for the three age subgroups (<65-, ≥65 to <75-year-old group, and ≥75-year-old group) [18], and we combined the HR of the two groups (≥65 to <75-year-old group and ≥75-year-old group) for further analysis. We use random-effect model due to the high heterogeneity (*I*^2^ = 65.6%). According to our analysis, a significantly prolonged PFS was demonstrated in younger (HR: 0.73, 95% CI: 0.61–0.85, *P* = 0.000) and older people (HR: 0.62, 95% CI: 0.49–0.76, *P* = 0.000) with a cut-off age of 65 (Figure 3). Subgroup analysis of PFS by the type of PD-1 inhibitors (nivolumab and pembrolizumab) demonstrated that efficacy of nivolumab was better than pembrolizumab in both younger and older groups (Table 2).

## DISCUSSION

### Main findings

To the best of our knowledge, this meta-analysis was the first study exploring the correlation between ages and NSCLC patients’ survival in PD-1 inhibitor treatment. Recent clinical trials suggested the possible effect of age on the effectiveness of PD-1 inhibitors. However, no solid conclusions were made, partly due to the lack of statistical power. Hence, the aim of this study was to verify our hypothesis and provide practical suggestions in NSCLC treatments. We found that, with a cut-off age of 65, PD-1 inhibitors were associated with significantly longer OS and PFS in younger and older groups versus chemotherapy. Also, among NSCLC patients aged over 75, no prolonged OS was observed compared with controls. In addition, older patients treated with nivolumab have a better survival (OS and PFS) in comparison with pembrolizumab, and, among younger patients in treatment of nivolumab, a longer OS was observed as opposed to pembrolizumab. The observed results may not be entirely applicable to all patients treated in the community.

### Agreement/disagreement with previous studies and interpretations

The age-related changes in the innate and adaptive immune system may have varying clinical consequences at different levels, and published reviews have described this phenomenon without a quantifying analysis [12, 19, 20].

Immunosenescence is a physiological process causing alterations of immune functions resulting from age. Researches on the role of immunosenescence in tumourigenesis provided insights into the possible rationale of age-related reduced efficacy. It was observed that immunosenescence is associated with impaired T cell activation, reduced tumour antigen release available for processing by antigen presenting cells, and reduced ability of T cells to eliminate tumor cells [12]. Interestingly, previous study showed that T cells exhibit senescent changes after 70 years of age [21], which is partly in line with our results. Also, reduced responsiveness of immune cells to cytokines (IL-2 and IL-12) and chemokines, which correlates with reduced trafficking and proliferation, was caused by the increasing age. This may lead to a decrease in the release of neo-antigens, thus impairing the initiation of antitumor immune responses [22]. Notably, CD8+ T cells were the major mediator influenced by the PD-1 ligand pathway, and aging is associated with a decreased compartment of naïve CD8+ T cells [23]. This preclinical finding, in the context of cancer, may partly account for our clinical results. A dialogue between preclinical researchers and clinicians is required for the better understanding and application of PD-1 inhibitors.

Latest clinical studies manifested a possible effectiveness-age correlation in PD-1 inhibitor trials. Scientists in CheckMate 017 trial reported that the HRs of OS did not favored nivolumab in patients aged over 75 years old, which is consistent with our results. Researchers in this trial did not draw a final conclusion, partly attributing it to small sample sizes. Our study, with a extended scope and larger numbers of patients included, reinforced their finding. Another meta-analysis compared the efficacy of different ICIs in varying cancer types in general, however, the absence of specific analysis for NSCLC patients prevented a practical NSCLC guideline for clinicians.

### Limitations

Some limitations of the present meta-analysis should be acknowledged. A major concern was that we include patients with different types of NSCLC with different etiologies and disease courses. We have minimized its influence by using the random-effects model when the heterogeneity was high. Also, subgroup analysis was based on relatively small sample sizes, and those data required further evaluation in prospective randomized clinical trials. Especially in the group of patients over 75 years old, the outcome was based on a comparatively small sample size, although the results were in accordance with a former study [8]. Our study only include four RCTs, which may cause the unobserved heterogeneity. This may partly lead to some uncertainties of outcomes. In the future, a potential meta-analysis identified was comparison on toxicity of ICIs between males and females, although valid data are currently absent. The preclinical mechanism is worth further investigations.

## CONCLUSIONS

With a cut-off age of 65, significantly longer overall survival and progression-free survival were observed in younger and older NSCLC patients treated with PD-1 inhibitors versus chemotherapy. Among patients aged over 75, no significantly prolonged overall survival was observed compared with chemotherapy. In comparison with pembrolizumab, nivolumab was associated with better overall survival in the older NSCLC patients (≥65-year-age), and better progression-free survival in both younger and older people. Older patients, especially those aged over 75, should be paid more attention to in the future clinical trials, guidelines, and clinical practice.

## METHODS

### Study design and search strategy

This meta-analysis was based on data from RCTs, which compared PD-1 inhibitors with chemotherapies in younger and older NSCLC patients. We searched for trials on PubMed/Medline, Embase, the Cochrane Central Register of Controlled Trials (CENTRAL), and Google Scholar; September 2017 was the cut-off date. Key words included PD-1 inhibitor, pembrolizumab, nivolumab, age, and clinical trial. All the studies of potential interest were retrieved.

### Selection criteria

Eligible studies should fulfill the following criteria. (1) Trials that included patients with NSCLC. (2) Interventions of PD-1 inhibitors (pembrolizumab and nivolumab). (3) Trials that compared PD-1 inhibitors and chemotherapies. (4) Trials that reported Hazard ratios (HRs) of overall survival (OS) or progression-free survival (PFS) and subgroup analysis by age. (5) Phase III RCTs. Trials would be excluded if the control group is ICI immunotherapy. All the articles that were not published in English were excluded.

### Data extraction

Three reviewers (YW, FZ, HS) independently extracted data with a piloted extraction form, and checked all the data very carefully. We identified all studies by first author and the year of publication, and extracted the following information from the reports: Year of publication, inclusion or exclusion criteria, sample size, drugs and doses in the experimental groups and control groups, HR of PFS or OS in younger and older patients. The primary outcome measured in the meta-analysis was the OS, and secondary outcome was the PFS. All the duplicated studies were excluded.

### Risk of bias assessment

Three reviewers (YW, FZ, HS) independently assessed the risk of bias using the Cochrane Collaboration tool. All disagreements were resolved by the first author (YW). Details on the risk of bias in eight studies are illustrated in Supplementary Figure 2.

### Statistical analysis

Statistical analysis, forest plots, and detection of publication bias were done with Stata SE 14 (StataCorp, College Station, TX, USA) for macOS Version 10.12.5. Risk of bias was calculated using Review Manager (RevMan5.3; The Nordic Cochrane Centre, The Cochrane Collaboration, Copenhagen, Denmark). HRs were used for evaluation and 95% confidence intervals (CIs) were calculated for each estimate. *P* ≤ 0.05 was considered statistically significant. Heterogeneity was considered low, moderate or high for *I*^2^ values <25%, 25–50%, and >50%, respectively [14]. If the *I*^2^ value was lower than 50%, A fixed model would be used; if the *I*^2^ value was more than 50%, A random model would be used. Publication bias was assessed by both Begg's test and Egger's test.

## SUPPLEMENTARY MATERIALS FIGURES



## Abbreviations

APC: antigen-presenting cell
ICI: immune checkpoint inhibitor
NSCLC: non-small cell lung cancer
OS: overall survival
RCT: randomized controlled trial
PD-1: programmed death-1
PFS: progression-free survival.
