# Supplementary material for: The effectiveness of PD-1 inhibitors in non-small cell lung cancer (NSCLC) patients of different ages
Source: Oncotarget. 2017 Dec 26;9(8):7942–8. doi: 10.18632/oncotarget.23678 (PMC5814271; doi:10.18632/oncotarget.23678)
Supplement: Supplementary file 1 [file oncotarget-09-7942-s001.pdf]

The effectiveness of PD-1 inhibitors in non-small cell lung cancer (NSCLC) patients of different ages

SUPPLEMENTARY MATERIALS

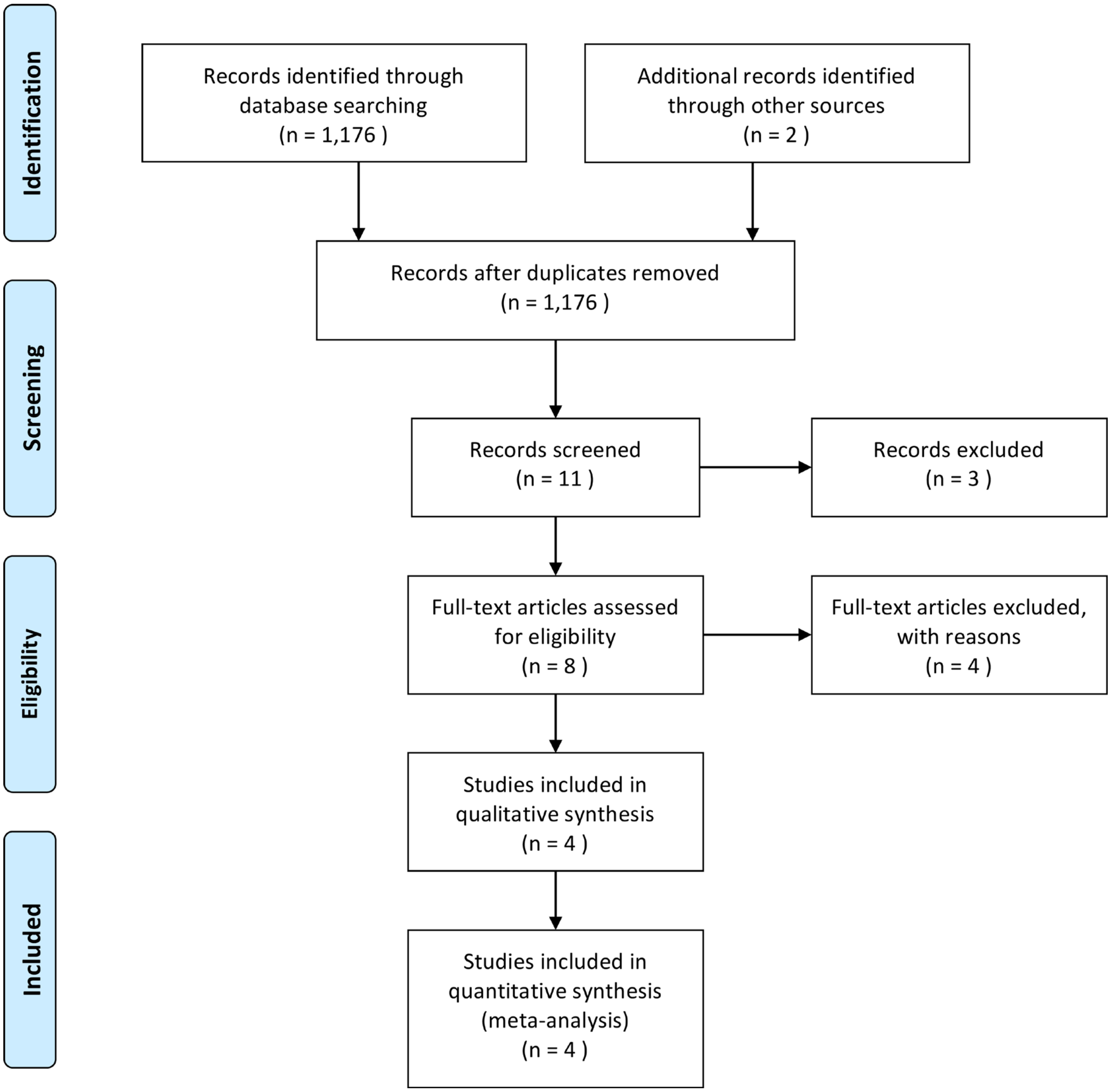

Supplementary Figure 1: The process of trial selection.

	Random sequence generation (selection bias)	Allocation concealment (selection bias)	Blinding of participants and personnel (performance bias)	Blinding of outcome assessment (detection bias)	Incomplete outcome data (attrition bias)	Selective reporting (reporting bias)	Other bias
Borghaei 2015	+	?	−	+	+	+	+
Brahmer 2015	+	?	−	+	+	+	+
Herbst 2016	+	?	+	?	+	+	+
Reck 2016	+	?	−	?	+	+	+

Supplementary Figure 2: Quality assessment of the included studies.
